# Pelvic hemorrhage in the context of demographic change: frequency and CT-based characterization of pelvic bleeding

**DOI:** 10.1007/s00068-026-03363-6

**Published:** 2026-10-15

**Authors:** Florentine Gerhardt, Andreas Höch, Dmitry Notov, Jeanette Henkelmann, Suzanne Zeidler, Stephanie Einhorn, Christian Kleber

**Affiliations:** 1 Department of Orthopaedic, Trauma and Plastic Surgery, Leipzig University Hospital Leipzig, Leipzig, Germany; 2 Department of Diagnostic and Interventional Radiology, Leipzig University Hospital Leipzig, Leipzig, Germany

**Keywords:** Pelvic fracture, Pelvic hemorrhage, Contrast-enhanced computed tomography, Fragility fractures of the pelvis, Hemorrhage

## Abstract

**Purpose:**

Traumatic pelvic hemorrhage is a major driver of morbidity and mortality in pelvic fractures. Classic literature described pelvic hemorrhage as predominantly venous, whereas arterial injury is considered less common. We evaluated the frequency, anatomical distribution, and presumed sources of pelvic hemorrhage in a contemporary trauma cohort using contrast-enhanced CT (CECT).

**Methods:**

We conducted a single-center, retrospective cohort study of consecutive patients with pelvic fractures treated at a level-I trauma center between 2020 and 2022. All patients underwent CECT at initial presentation. Two experienced pelvic trauma surgeons independently reviewed all scans. Radiologically relevant hemorrhage was defined by contrast extravasation, soft-tissue asymmetry/displacement, or a contained intrapelvic hematoma. Patients with and without hemorrhage were compared regarding age, sex, trauma mechanism, anticoagulation, and fracture type. The presumed bleeding sources were categorized as arterial, venous, cancellous, or mixed.

**Results:**

Radiologically relevant hemorrhage occurred in 95/512 (18.6%) patients and was more frequent after high-energy trauma and in men. Iliac sites predominated, whereas presacral bleeding was uncommon. The presumed bleeding source was cancellous in 44.2%, mixed in 29.5%, arterial in 18.9%, and isolated venous in 7.4%. Overall mortality was 7.0% and was significantly higher in patients with hemorrhage (14.7% vs. 5.3%; *p* < 0.001).

**Conclusion:**

In this contemporary CECT-based cohort, pelvic hemorrhage was less frequent than historically reported, with CT findings most commonly suggesting cancellous or mixed bleeding sources. These findings suggest that the traditional concept of predominantly venous pelvic hemorrhage may not fully reflect current CT-based observations.

## Introduction

Traumatic pelvic hemorrhage is a potentially life-threatening complication and remains a major contributor to morbidity and mortality in patients with pelvic fractures, particularly in the setting of polytrauma [1, 2]. Due to the complex pelvic anatomy and the limited ability for spontaneous tamponade, uncontrolled hemorrhage may rapidly lead to hemodynamic instability and death if not promptly recognized and treated [3].

For decades, the prevailing concept in trauma surgery has been that pelvic bleeding predominantly originates from venous structures, most notably the presacral venous plexus and disrupted cancellous bone surfaces. Classical studies have reported venous bleeding as the primary source in up to 80–90% of cases, whereas arterial injuries were considered comparatively rare, accounting for only 10–20% [4–6]. These assumptions have strongly influenced contemporary diagnostic algorithms and therapeutic strategies, emphasizing external fixation, pelvic packing, and venous tamponade as first-line measures [7].

However, much of the existing literature is based on heterogeneous trauma populations, historical cohorts, or data predating modern imaging techniques. In recent years, demographic changes have led to a substantial increase in elderly trauma patients with pelvic fractures, often resulting from low-energy mechanisms and frequently associated with osteoporosis and comorbidities [8–10, 29]. At the same time, advances in computed tomography have significantly improved the detection and characterization of pelvic hemorrhage, allowing for a more detailed assessment of bleeding patterns and sources [11].

Emerging evidence and clinical observations at our center suggest that the traditional paradigm of predominantly venous pelvic bleeding may no longer adequately reflect contemporary trauma populations. Modern imaging studies have indicated a higher prevalence of arterial and osseous bleeding sources than previously assumed, particularly in elderly patients and in specific fracture patterns [12–14]. Nevertheless, data remain limited, and the true distribution and origin of pelvic hemorrhage in contemporary trauma populations are still incompletely understood. Furthermore, studies focusing on angiographic embolization are inherently prone to selection bias, potentially overestimating the prevalence of clinically relevant arterial hemorrhage in pelvic trauma [23]. Therefore, the aim of the present study was to critically re-evaluate the frequency, anatomical distribution, and presumed sources of pelvic bleeding in patients with pelvic fractures in the context of an ageing trauma population using CT-based assessment. By comparing patients with and without radiologically relevant pelvic hemorrhage, we sought to identify factors associated with bleeding occurrence and to assess the traditional concept of pelvic hemorrhage in light of contemporary CT findings.

## Study design and patients

The local institutional ethics committee approved this retrospective cohort study (reference no. 043/23-ek). A total of 512 consecutive patients with a pelvic fracture following any type of trauma were included. All patients were admitted to a level I university trauma center between January 2020 and December 2022 and had undergone contrast-enhanced computed tomography (CECT) imaging of the pelvis at initial presentation.

We identified patients using the institutional pelvic fracture database. Patients without CECT imaging of the pelvis or with incomplete radiological data sets were excluded from further analysis.

### Radiological assessment and definition of pelvic bleeding

All available CECT scans were retrospectively reviewed independently by two experienced pelvic surgeons. In addition, we reviewed the corresponding radiological reports and considered them during final classification. 

Radiologically relevant pelvic hemorrhage was defined by the presence of at least one of the following criteria:

- active contrast extravasation,
- a contained intra-pelvic hematoma.
- or asymmetrical displacement of pelvic soft tissues suggestive of hematoma formation.

Because these findings represent complementary radiological signs of pelvic hemorrhage, the presence of at least one criterion was considered sufficient for classification as radiologically relevant hemorrhage. In patients with radiologically detectable hemorrhage, the presumed predominant bleeding source was categorized as arterial, venous, cancellous osseous, mixed, or indeterminate. This classification was based on the morphology and temporal appearance of contrast extravasation, the hematoma's anatomical distribution of the hematoma, and its spatial relationship to identifiable vessels and fracture surfaces.

- Arterial bleeding was presumed when focal or linear contrast extravasation was visible and could be anatomically related to an arterial vessel or vascular territory. A focal contrast blush with an attenuation comparable to that of the arterial blood pool was also considered suggestive of an arterial source.
- Venous bleeding was presumed when diffuse or delayed contrast extravasation was present without a clearly identifiable arterial origin and when the hemorrhage was located along typical pelvic venous structures or plexuses, particularly in the presacral or perivesical regions.
- Cancellous osseous bleeding was presumed when a hematoma was located immediately adjacent to a fracture surface or within the surrounding soft tissues without visible contrast extravasation from an identifiable arterial or venous structure. This classification required direct spacial relationship between the hematoma and the cancellous fracture surface.
- Mixed bleeding was presumed when imaging findings suggested more than one bleeding source, for example, arterial contrast extravasation combined with extensive hemorrhage directly related to cancellous fracture surfaces or venous plexuses.

### Data collection

For all patients, demographic and clinical variables were collected, including age, sex, and trauma mechanism, which was categorized as high-energy or low-energy trauma. Fractures were classified according to the AO/OTA classification, the Judet–Letournel classification for acetabular fractures, as well as the Fragility Fractures of the Pelvis (FFP) classification.

In addition, the intake of anticoagulant medication was documented and categorized as antiplatelet agents, direct oral anticoagulants (DOACs), vitamin K antagonists or heparin.

### Subgroup analysis of patients with pelvic bleeding

Based on the predefined radiological criteria, 95 out of 512 patients (18.6%) were identified as having radiologically relevant pelvic bleeding. These patients were statistically compared with the overall pelvic fracture cohort to identify potential risk factors for pelvic bleeding. The predefined hypothesis was that trauma mechanism, fracture classification, and the use of anticoagulant medication are associated with an increased risk of pelvic bleeding.

In patients with radiologically relevant hemorrhage, the presumed source of bleeding was further classified as arterial, venous, cancellous (osseous), or mixed bleeding. Furthermore, it was recorded whether patients were admitted via the resuscitation room, as well as their initial hemodynamic status and hemoglobin level at hospital admission.

### Statistical analysis

All data were recorded in a Microsoft Excel database (Microsoft Corp., Washington, DC, USA) and subsequently exported to SPSS Statistics version 29.0 (IBM Corp., Chicago, IL, USA) for statistical analysis. Histogram analysis demonstrated a normal distribution of continuous variables. Data are presented as frequencies and means ± standard deviation.

Student’s t test was used to compare differences in means between groups. Categorical variables were analyzed using contingency tables and Pearson’s chi-squared test or Fisher’s exact test, as appropriate. A p value < 0.05 was considered statistically significant.

## Results

Of the 512 patients with pelvic fractures included in this study, 95 patients (18.6%) demonstrated radiologically relevant signs of pelvic hemorrhage on initial CT imaging.

### Patient demographics and trauma characteristics

Patients with pelvic bleeding had a mean age of 70 ± 17.6 years, compared to 68 ± 22.0 years in patients without bleeding. There was no statistically significant difference in age between groups. A statistically significant male predominance was observed in the bleeding cohort (55 vs. 42%; *p* = 0.018).

High-energy trauma was significantly more frequent in patients with pelvic bleeding (73% vs. 48%, *p* < 0.01) **(**Table 1**).**

**Table 1:** Patient demographics and trauma characteristics

Variable	Total cohort (*n* = 512)	Bleeding (*n* = 95)	No bleeding (*n* = 417)	*p* value
Age, years (mean ± SD)	68 ± 21.3	70 ± 17.6	68 ± 22.0	0.343
Male sex, n (%)	224 (43.8)	52 (54.7)	172 (41.2)	0.017
High-energy trauma, n (%)	270 (52.7)	69 (72.6)	201 (48.2)	< 0.01

### Fracture patterns

Within the overall cohort, pelvic fracture patterns were heterogeneously distributed. According to the AO/OTA classification, type B2 injuries were the most common fracture pattern, accounting for 24% (*n* = 121) of all cases, followed by isolated acetabular fractures without concomitant pelvic ring injury in 21% (*n* = 105) and type C1 pelvic ring fractures in 12% (*n* = 63). Further relevant fracture types included AO type A2 fractures in 11% (*n* = 57), type B1 fractures in 7% (*n* = 34), and type C3 fractures in 6% (*n* = 32).

Among patients with isolated acetabular fractures, fractures of the anterior column combined with a posterior hemitransverse component were most frequent, occurring in 31% (*n* = 37) of cases. This was followed by isolated anterior column fractures in 25% (*n* = 30) and both-column fractures in 22% (*n* = 27). All remaining acetabular fracture patterns accounted for less than 7% each.

Combined injuries involving both the pelvic ring and the acetabulum were observed in 15 patients (3% of the overall cohort).

In patients with radiologically relevant pelvic bleeding, one third of bleeding events were associated with either acetabular fractures or AO type C pelvic ring injuries.

In the overall cohort, FFP (i.e., low-energy fragility injuries) accounted for 47.3% (*n* = 242). Within the FFP spectrum, type II lesions predominated, with FFP IIb most frequent (26.9%, *n* = 65), followed by FFP IVb (23.6%, *n* = 57), FFP Ia (14.0%, *n* = 34) and FFP IIc (12.8%, *n* = 31). Less common were FFP IIIa and FFP IVa (each 7.4%, *n* = 18) and FFP IIa (5.8%, *n* = 14); all other subclasses were rare (≤ 1% each). The remaining 52.7% (270/512) were non-FFP/high-energy injuries.

Bleeding clustered in FFP IIb and FFP IIIa (each 30.8%, 8/26), followed by FFP IIc (19.2%, 5/26); single cases occurred in FFP Ia, FFP Ib, FFP IIa and FFP IVa (each 3.8%, 1/26), with no events in FFP IIIb/IVb/IVc.

### Bleeding rates according to fracture classification

**Fig. 1:**
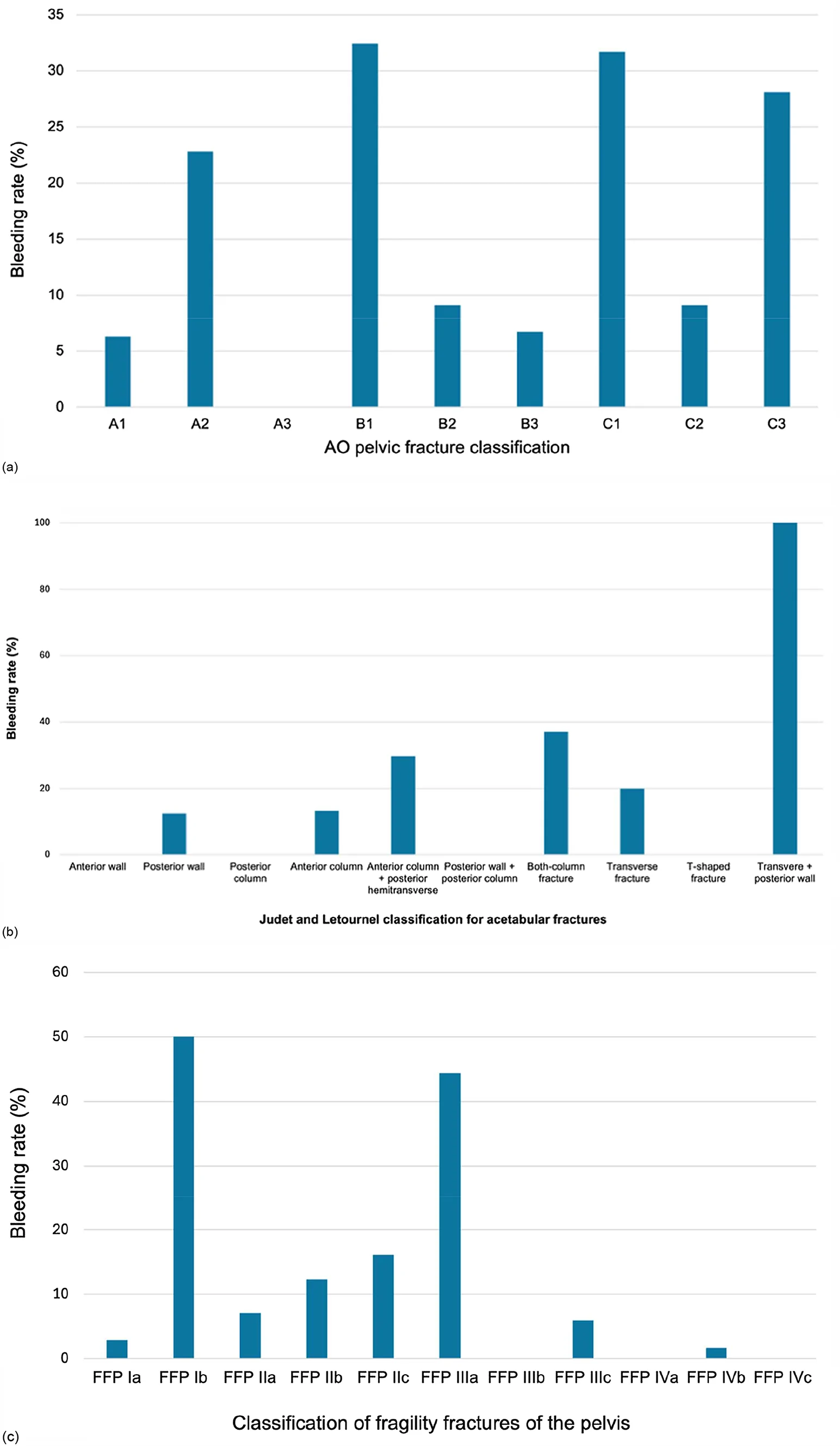
Bleeding rates according to pelvic fracture classification. (**a**) AO/OTA: rates are lowest in stable A, increase in rotationally unstable B (notably B2), and are highest in vertically/combined unstable C (C1–C2); (**b**) Judet–Letournel: patterns with posterior column/transverse/associated involvement exceed simple anterior patterns; (**c**) FFP: rates rise with instability from FFP I (low) through FFP II (intermediate) to FFP III–IV (highest)

Bleeding rate denotes the pattern-specific proportion of patients with radiologically relevant signs of pelvic hemorrhage (bleeding cases ÷ all cases within that fracture pattern), i.e., a risk measure rather than a prevalence measure **(**Fig. 1**).**

### Anticoagulation

Most patients with pelvic bleeding (67.3%) were not receiving anticoagulant therapy at the time of injury. Anticoagulant use did not differ statistically between patients with and without bleeding (Table 2).

**Table 2:** Anticoagulant medication at time of injury

Medication	Bleeding (*n* = 95)	No bleeding (*n* = 417)	*p* value
No anticoagulation	64 (67.3%)	285 (68.3%)	*p* = 0.479
DOAC	18 (18.9%)	66 (15.8%)	
Antiplatelet therapy	10 (10.5%)	52 (12.5%)	
Vitamin K antagonist	3 (3.2%)	7 (1.7%)	
Heparin	0 (0.0%)	7 (1.7%)	

### Origin of bleeding

**Fig. 2:**
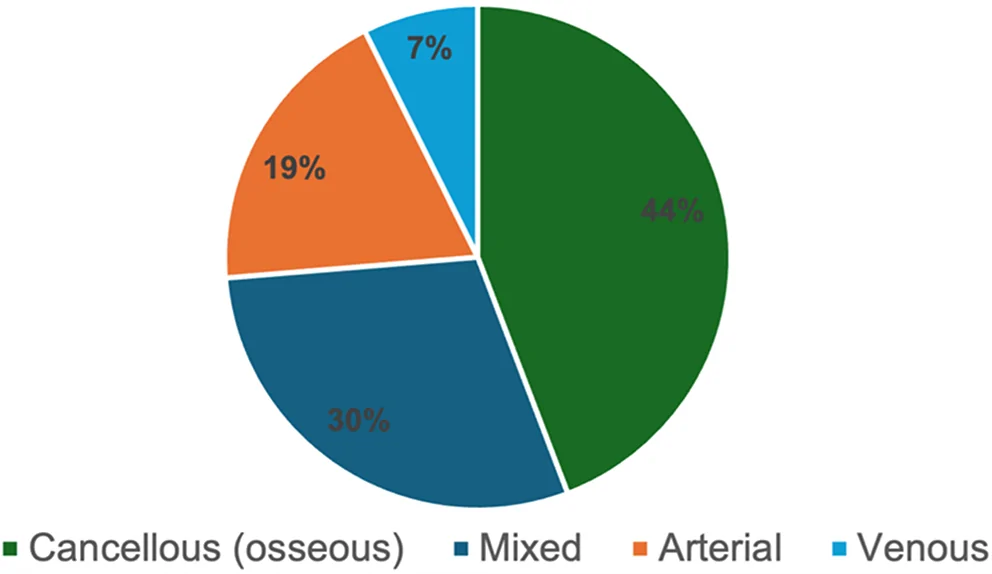
Origin of pelvic bleeding (*n* = 95). Distribution of presumed bleeding sources among patients with radiologically relevant pelvic hemorrhage (*n* = 95)

Among patients with radiologically relevant pelvic hemorrhage, the most frequent presumed bleeding source was cancellous (44.2%), followed by mixed bleeding sources (29.5%). Arterial bleeding was identified in 18.9% of cases, whereas isolated venous bleeding occurred in only 7.4% **(**Fig. 2**).**

### Mortality

Overall mortality in the study cohort was 7.0%. Mortality was significantly higher in patients with pelvic bleeding compared to those without bleeding (14.7% vs. 5.3%, *p* < 0.001).

## Discussion

Traumatic pelvic hemorrhage remains one of the most critical challenges in acute trauma care and continues to be associated with substantial morbidity and mortality. The present study provides a contemporary, CECT-based reassessment of the frequency, anatomical distribution, and presumed sources of pelvic bleeding in patients with pelvic fractures, with a particular focus on the impact of demographic change and evolving fracture patterns.

In the present cohort, radiologically relevant pelvic bleeding was identified in 18.6% of patients with pelvic fractures. This incidence is notably lower than that reported in several historical series, which often described pelvic hemorrhage as a frequent and almost ubiquitous complication in unstable pelvic injuries [1, 2, 5, 17]. These discrepancies likely reflect differences in study design, patient selection, and diagnostic methodology. Earlier studies frequently relied on clinical surrogates, intraoperative findings, or postmortem analyses, whereas the present study applied strict CECT-based criteria to define relevant bleeding [4].

One of the most important findings of this study is the predominance of osseous (cancellous) bleeding, accounting for more than 40% of all bleeding events. In contrast, isolated venous bleeding from the presacral plexus was detected in only a small proportion of patients. This observation directly challenges the long-standing paradigm that pelvic hemorrhage is predominantly venous in origin, a concept derived mainly from postmortem and early surgical studies [4, 5]. More recent investigations have already questioned this paradigm and suggested a more heterogeneous distribution of bleeding sources, which is strongly supported by the present data [13, 18, 22].

Arterial bleeding was identified in approximately one fifth of patients with pelvic hemorrhage. Although arterial injuries were traditionally considered rare, recent studies have demonstrated a higher prevalence of arterial bleeding, particularly in selected patient populations [12, 13, 22]. In the present cohort, arterial bleeding was more frequently observed in elderly patients following low-energy trauma, indicating a distinct bleeding pattern in fragility fractures of the pelvis. This finding is in line with previous reports emphasizing the vulnerability of elderly patients to arterial injury even after low-energy mechanisms [14, 25, 28].

In our cohort, bleeding was more often mapped to the iliac wing, whereas presacral sources were comparatively uncommon. This distribution may reflect a contemporary patient population—characterized by more low-energy fragility injuries and acetabular fractures [8–10, 25, 27]—together with improved CT-based spatial localization and hematoma characterization [11,16, 21].

The association between pelvic bleeding and increased mortality observed in this study is consistent with previous reports [1, 5, 21]. However, the overall mortality rate was lower than that reported in many historical cohorts, which may reflect improvements in trauma systems, early whole-body CT imaging, and the availability of multimodal hemorrhage control strategies, including pelvic stabilization, preperitoneal pelvic packing, and angiographic embolization [15,18–20, 24].

Interestingly, anticoagulant medication was not significantly associated with the occurrence of pelvic bleeding. This suggests that fracture morphology and trauma mechanism may be more relevant determinants of bleeding risk than anticoagulation alone. Similar observations have been reported in studies focusing on geriatric pelvic trauma, where bone quality and fracture instability appear to outweigh pharmacological factors [14, 25, 26].

The findings of the present study may have relevant clinical implications. The predominance of osseous and mixed bleeding sources questions the traditional emphasis on venous tamponade alone and highlights the need for individualized diagnostic and therapeutic strategies. Among elderly patients undergoing CECT, CT-based characterization of pelvic hemorrhage may help guide individualized management [18, 20, 24].

This study has several limitations. First, its retrospective single-center design limits generalizability and may introduce selection bias. Second, bleeding sources were inferred from CECT findings and retrospective image review rather than systematically confirmed by angiography, surgery, or postmortem examination. Third, only patients who underwent contrast-enhanced CT at initial presentation were included. Therefore, patients who died at the scene, during prehospital care, or before imaging could be obtained were not captured, which may have led to an underestimation of the most severe and rapidly fatal pelvic hemorrhages. Fourth, exclusion of patients without CECT or with incomplete imaging datasets may have further affected cohort composition. Finally, although mortality was higher in patients with pelvic bleeding, the study design does not allow causal conclusions, and residual confounding by injury severity, comorbidities, and treatment factors cannot be excluded.

In a modern CECT-based trauma cohort, pelvic hemorrhage was less frequent than historically reported and was predominantly osseous or mixed rather than isolated venous in origin. These findings challenge the longstanding venous paradigm of pelvic bleeding and highlight the importance of early CT-based localization to guide individualized, multimodal hemorrhage control. This may be particularly relevant in contemporary trauma populations increasingly shaped by older patients and fragility-related pelvic fractures.

## Data Availability

The datasets generated and/or analyzed during the current study are not publicly available due to privacy and ethical restrictions but are available from the corresponding author on reasonable request.
